# Preparation of Shellac Resin Microcapsules Coated with Urea Formaldehyde Resin and Properties of Waterborne Paint Films for Tilia amurensis Rupr.

**DOI:** 10.3390/membranes10100278

**Published:** 2020-10-12

**Authors:** Xiaoxing Yan, Lin Wang

**Affiliations:** 1 Co-Innovation Center of Efficient Processing and Utilization of Forest Resources, Nanjing Forestry University, Nanjing 210037, China; 2 College of Furnishings and Industrial Design, Nanjing Forestry University, Nanjing 210037, China; wanglin@njfu.edu.cn

**Keywords:** microcapsules, shellac, waterborne paint films, performance

## Abstract

A two-step in situ polymerization method was utilized to fabricate urea formaldehyde (UF) resin-coated shellac resin microcapsules. The morphology and composition of microcapsules with different core-wall ratios were analyzed by scanning electron microscope (SEM) and infrared (IR) spectrum. The effects of different concentrations of microcapsules on gloss, color difference, hardness, adhesion, and impact resistance of waterborne paint films were studied. At the same time, the self-healing effect of the prepared microcapsules applied to waterborne paint film was discussed. The results revealed that the shellac resin microcapsules coated with UF resin were successfully prepared. At the 0.67:1 and 0.75:1 core-wall ratios, the color differences of the paint film with 0–20.0% (weight percent) microcapsules were small and the color was uniform. Under the condition of 60° incident angle and the same microcapsule concentration, a good gloss was obtained. When the concentration was 20.0%, the hardness of paint film reached the maximum value. The adhesion of paint film was better, which was not affected by microcapsule concentration. When the concentration was 5.0% and 10.0%, the microstructure of paint film was good. The paint film with a 10.0% concentration of the shellac resin microcapsules coated with UF resin had better self-healing performance and the comprehensive performance was better. This paper provides the basis for the industrial application of self-healing waterborne wood paint films.

## 1. Introduction

Microcapsule technology is a kind of technology used to store core materials (usually in the form of solid, liquid, and gas) in polymer wall materials [1]. The wall materials are composed of film-forming materials that are mainly natural or synthetic materials in a solid state [2]. A core material is a material coated with a wall material, which is the hinge to determine the function of microcapsules [3]. In self-healing paint films, the core material of microcapsules achieves the repair effect through external stimulation and slow release [4]. Therefore, the good fluidity, proper curing speed, and good compatibility with paint film substrate are the necessary conditions for selecting core materials [5]. Compared with the traditional repair technology, the key for microcapsule technology lies in its simple synthesis, low cost, and self-healing of materials without the aid of external conditions [6].

Xu et al. [7] prepared urea formaldehyde (UF) microcapsules to ameliorate the spontaneous cure property of coral sand cementitious materials. The study revealed that the optimization particle size distributions and volume fractions of microcapsules are 450 rpm and 3%. The results show that the spontaneous cure effect of microcapsules synthetized under this condition was about 75%. Sun et al. [8] prepared sunflower oil microcapsules to enhance the technical and self-repairing performance of bitumen-based sealants, which were added into the bitumen-based sealants. The self-healing properties of microcapsule sealant was obviously superior to the original sealant. Sunflower oil microcapsules could heighten the repeated self-repairing capability and fatigue life of the sealants at −20 °C. Mirabedini et al. [9] prepared polyurea-formaldehyde-based microcapsules loaded with linseed oil. The conclusions showed amelioration in tensile strength abilities and corrosion resistance via the simultaneous use of 3-aminopropyltrimethoxy silane (APS)-treated microcapsules and nanoclay. APS-treated microcapsules more easily ruptured during scratching, resulting in better corrosion resistance and self-repairing capabilities. The above studies all focus on the self-healing efficiency and anti-corrosion of coral sand cementitious materials, bitumen-based sealants, and nanocomposite paint film. However, the studies on the self-healing of waterborne wood paint films are few.

Shellac is a natural resin secreted by rubber insects after absorbing sap from host trees [10]. After removing impurities from the rubber block collected from the tree [11], it was crushed, screened, mixed, rinsed with water, and dried with a dryer to form shellac [12]. Shellac is a natural product, is non-toxic, provides environmental protection, and has good film-forming ability, fast drying and curing speed, unique solubility, and excellent thermal performance [13]. Shellac paint is also widely used in the field of wood furniture [14]. Shellac resin is selected as the repair agent which can realize room temperature curing [15]. UF resin is translucent after curing and has a certain level of toughness and strength [16,17]. Its preparation cost is low, and its raw materials are rich and its performance is stable [18]. Waterborne paint films have become newly popular in the territory of paint films as a result of their low or no volatile organic compounds (VOCs) and harmful air pollutants [19,20].

In the preliminary work [21], epoxy resin coated with UF resin microcapsules were fabricated. The morphology and structure of microcapsules were determined by orthogonal experiments with five factors and four levels. The yield, optimal repair rate, and coverage rate of microcapsules were analyzed. Nevertheless, once the microcapsule breaks, epoxy resin as a repair agent of microcapsules for paint films on wood substrate needs to be cured via heating or adding curing agent. This is obviously unrealistic for intelligent self-healing wood products.

In this paper, UF resin (wall material) and shellac (core material) were used to prepare microcapsules via in situ polymerization. The optimal core-wall ratio was determined by studying the effect of different core-wall ratios on microcapsules. Using *Tilia amurensis Rupr*. as a wood substrate, the effect of UF resin-coated shellac microcapsules on the mechanical and optical properties of waterborne paint films was studied. The self-healing effect of the prepared microcapsules on the *Tilia amurensis Rupr*. surface was observed by simulating the damage of the paint film in the process of day-to-day operations. This was a new attempt to realize self-healing waterborne paint films, which provided a new possibility for the application of microcapsule self-healing technology in the territory of wood furniture.

## 2. Materials and Methods

### 2.1. Test Materials

The main raw materials for preparing the shell of microcapsules are urea (M_w_: 60.06 g/mol, CAS No.: 57-13-6) and 37.0% formaldehyde solution (M_w_: 30.03 g/mol, CAS No.: 50-00-0). The pH regulators are triethanolamine (M_w_: 149.19 g/mol, CAS No.: 102-71-6) and citric acid monohydrate (CAM, M_w_: 210.14 g/mol, CAS No.: 5949-29-1). The emulsifier is sodium dodecyl benzene sulfonate (SDBS, M_W_: 348.48 g/mol, CAS No. 25155-30-0). The solvent for dissolving shellac is anhydrous ethanol (M_w_: 46.07 g/mol, CAS No.: 64-17-5). The above materials were offered by Qiming Chemical Co., Ltd., Shandong, China. Shellac resin was offered by Jinan Dahui Chemical Technology Co., Ltd., Shandong, China. Waterborne wood coating was offered by Akzo Nobel Swire Paint Co., Ltd., Shanghai, China. Waterborne wood coating was composed of waterborne acrylic copolymer dispersion, matting agent, additive, and water. The solid matter of waterborne coating exceeded 30.0%. *Tilia amurensis Rupr*. (100.0 mm × 65.0 mm × 5.0 mm) was offered by Shantou Yihua Life Technology Co., Ltd., Guangdong, China. All reagents used in the experiment were not further treated.

### 2.2. Preparation of Microcapsules

The core-wall ratios of microcapsules were 0.42:1, 0.67:1, and 0.75:1, respectively. The materials for preparing microcapsules are exhibited in Table 1. The mass of core material was 12.5 g, 20.0 g, and 22.5 g, respectively, and the wall material quality remained unchanged. Three kinds of UF resin-coated shellac resin microcapsules were prepared. The preparation process of microcapsules with a 0.42:1 core-wall ratio was as follows:

Synthesis of UF prepolymer for wall material: 20.0 g urea and 27.0 g of 37.0% formaldehyde solutions were mixed thoroughly by stirring. The pH was adjusted to 8.0–9.0 via adding triethanolamine gradually. A magnetic stirring rotor was added and the mixture was mixed continuously in a 70 °C temperature water bath for 1 h, then the obtained emulsion was a wall material solution. The prepared wall material solution was naturally cooled to room temperature for use.

Synthesis of shellac resin core material emulsion: 62.5 g anhydrous ethanol and 12.5 g shellac resin were added into a beaker and stirred until completely dissolved. The 0.975 g SDBS white powder was added to 96.52 mL distilled water, stirred evenly, and 1.0% SDBS emulsifier solution was obtained. The emulsifier solution was added into the dissolved shellac solution and the magnetic stirring rotor was added for magnetic stirring. The uniform core emulsion was obtained after continuous stirring for 30 min at 1200 rotations per minute (rpm).

Microencapsulation: at the speed of 300 rpm, the cooling UF prepolymer was dropped into the core material emulsion, the CAM was added, and the pH was adjusted to 2.5–3.0. After the reaction was finished, the product was aged for 7 days and then washed and filtered by deionized water and anhydrous ethanol repeatedly. Finally, the residual product was dried at 80 °C for 4 h, and the resulting yellow powder was the microcapsules. The fabrication technique of microcapsules with core-wall ratios of 0.67:1 and 0.75:1 was the same as above.

### 2.3. Preparation of Paint Films

Three grams of waterborne primer were weighed in a beaker, and the substrate was evenly coated with waterborne primer once along the direction of the substrate texture with an SZQ tetrahedral fabricator (Guojing Electronic Co., Ltd., Jiangsu, China). The waterborne primer was placed in the front of the fabricator. The fabricator slid at a speed of 150 mm/s to obtain the required thickness of the coating. The film was first dried at room temperature for 30 min and then at 35 °C for 30 min. After drying, the paint film was polished with 800 mesh sandpaper, and then the dust was wiped off with a dry cloth. The above process is the process of applying the primer once, and the primer needs to be applied twice. Therefore, the above process needs to be repeated once more before the primer coating is completed. Three kinds of core-wall ratios (0.42:1, 0.67:1, and 0.75:1) of microcapsules were added to the topcoat to fabricate a self-healing topcoat with microcapsule concentrations of 0, 5.0%, 10.0%, 15.0%, and 20.0%. The composition is shown in Table 2. The topcoat was applied on *Tilia amurensis Rupr*. twice. The drying and grinding process of the topcoat paint film was the same as the primer. The thickness of all the obtained waterborne paint films was approximately 60 µm.

### 2.4. Performance Test

A conical hole with a sharp angle of 120 °C was drilled in the coating. The wall of the hole was imaged with a microscope magnified by 40 times. The length of the coating part of the bus bar was measured. Then, in accordance with the trigonometric function, the coating thickness was half of the bus coating length. The thickness of waterborne paint film was evaluated by the three-point arithmetic average method.

An HP-2136 portable colorimeter (Hanpu Testing Instrument Co., Ltd., Shenzhen, China) was used to detect the chroma value of paint film. The 60° gloss of the film was measured by an HG268 glossmeter (3NH Technology Co., Ltd., Shenzhen, China). Due to the wet expansion and dry shrinkage of wood, it easily cracks under high temperature. Therefore, the maximum temperature of 140 °C was selected as the limit value of thermal aging [22]. In order to simulate the aging phenomenon of paint films after long-term use, the coatings were heated at 120 °C for 7 h and then heated to 140 °C for 7 h. The color value, gloss, and stability of the film before and after aging were tested.

The film hardness was measured by a 6H-6B pencil (Dongguan Huaguo Precision Instrument Co., Ltd., Dongguan, China). The adhesion was evaluated by a QFH-HG600 film classifier (Yueqing Liushi Li Chuang Measurement Equipment Firm, Zhejiang, China). When the adhesion grade of the paint film was 0, the adhesion was the best. The impact resistance was evaluated by a QCJ impactor (Yemao Instrument Co., Ltd., Shanghai, China). Impact resistance refers to the maximum fall height to the test plate without causing film cracking.

The morphology of microcapsules and coatings was analyzed by a Quanta 200 environmental scanning electron microscope (SEM) (FEI company (Hillsboro, OR, USA)). The composition of the film was analyzed by a Vertex 80 V infrared spectrophotometer (Germany Bruker Co., Ltd., Karlsruhe, Germany). All experiments were repeated four times, and the error was less than 5.0%.

## 3. Results and Discussion

### 3.1. Microstructure and FTIR Analysis of Microcapsules with Different Core-Wall Ratios

**Figure 1:**
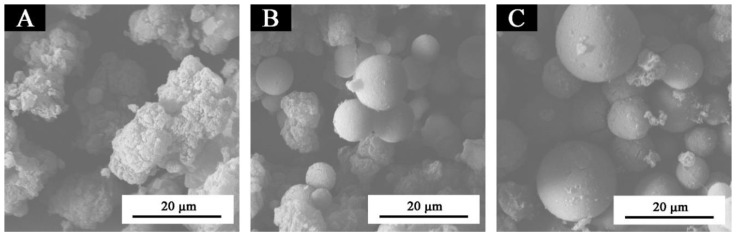
SEM of microcapsules with different core-wall ratios: (**A**) 0.42:1, (**B**) 0.67:1, (**C**) 0.75:1.

The microstructure of microcapsules is shown in Figure 1. As shown in Figure 1A, almost all microcapsules with a 0.42:1 core-wall ratio were amorphous particles, with large agglomerations and no spherical substances. Therefore, at the 0.42:1 core-wall ratio, the microencapsulation was not ideal. As shown in Figure 1B, two or three spheres agglomerated in the microcapsule with a 0.67:1 core-wall ratio. The spheres were relatively regular and the microcapsule surface was smooth, but there were still some amorphous particles. It can be seen from Figure 1B that the amorphous particles and the spheres coexist. As exhibited in Figure 1C, the microcapsules were regular spheres, with smooth and dense surfaces and slightly different particle sizes. The size of microcapsules was about 8.0–10.0 μm, and the spherical distribution was relatively uniform. Compared with the microcapsules with a 0.42:1 core-wall ratio in Figure 1B, the microcapsules with a 0.75:1 core-wall ratio in Figure 1C had a better microencapsulation degree and fewer amorphous particles. In conclusion, it can be preliminarily determined by SEM that the optimal core-wall ratio was 0.75:1.

**Figure 2:**
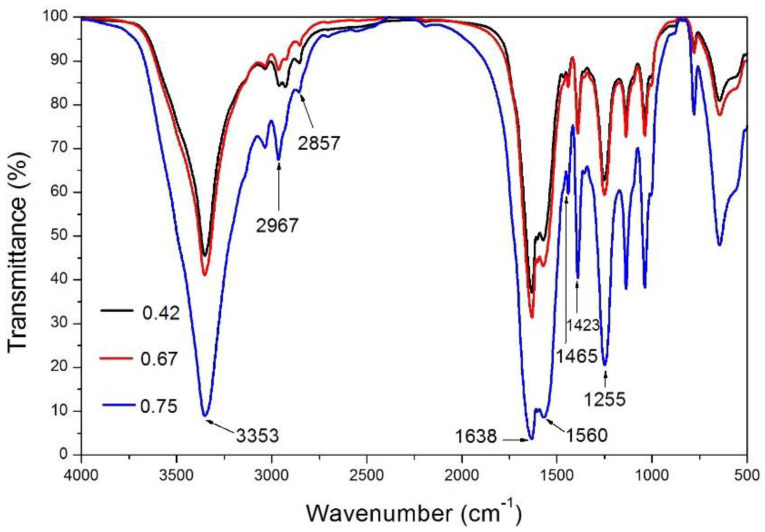
FTIR of microcapsules with different core-wall ratios.

Figure 2 shows the effect of microcapsules on the infrared spectrum of waterborne paint films. There was obvious absorption near 3353 cm^−1^, 2967 cm^−1^, 1638 cm^−1^, and 1560 cm^−1^, which were stretching vibrations of N–H, C–H, C=O, and C–N, respectively. According to the above four absorptions, the existence of UF resin as wall material was confirmed. Shellac resin is linked by ester bonds and contains hydroxyl and carboxyl groups [23]. The absorption of carboxyl hydroxyl vibration appeared at 3200–3500 cm^−1^, which coincided with the C–H absorption of UF resin, C–H absorption at 2857 cm^−1^, and three characteristic peaks of shellac appeared near 1465 cm^−1^, 1423 cm^−1^, and 1255 cm^−1^. Because the shellac was dissolved in alcohol and became liquid, the uncoated shellac would have been removed in the suction filtration process of microencapsulation, and there was no phenomenon of shellac monomer mixing in the microencapsulated powder. The shellac existed in the microcapsules and the chemical structure was not damaged. Consequently, the shellac was favorably coated by UF resin in the microcapsules with three core-wall ratios of 0.42:1, 0.67:1, and 0.75:1.

### 3.2. Effect of Microcapsule Concentration on Color Difference and Gloss of Paint Films

When multi-color light was applied as the light source, the color difference was the color difference of the paint films, which represented the difference degree of the overall brightness and color phase of the paint films. L is the lightness, a large value expresses that the color of the tested object surface is bright, and a small value indicates that the color of the tested object is dark. The a value expresses the variation of color from red to green. A positive value indicates red color and a negative value indicates green color. The b value expresses the variation of color from yellow to blue. A positive value indicates that the color of the tested object surface is yellow, and a negative value expresses blue. The c value is the color saturation. The H value is the hue. ΔL, Δa, and Δb were the variation values of black-white, red-green, and yellow-blue, respectively. L_1_, a_1_, and b_1_ represent the color values of a certain point of the paint films, while L_2_, a_2_, and b_2_ represent the color values of other points of the film. ΔL = L_1_ − L_2_, Δa = a_1_ − a_2_, Δb = b_1_ − b_2_. Therefore, the color difference can be calculated from Equation (1), as shown in Table 3, Table 4 and Table 5:

ΔE = [(ΔL)^2^ + (Δa)^2^ + (Δb)^2^]^1/2^ (1)

**Figure 3:**
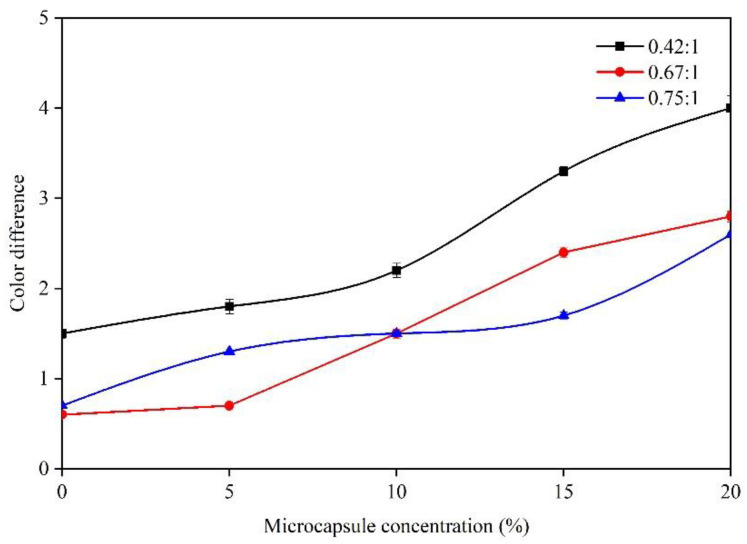
Effect of microcapsule concentration (0.42:1, 0.67:1, and 0.75:1 core-wall ratios) on color difference.

It can be seen from Figure 3 and Table 3, Table 4 and Table 5 that with the augmentation of microcapsule concentration, the color difference generally shows an upward trend, that is, the more microcapsules are added, the more uneven the color of the paint film is. At a 0.42:1 core-wall ratio, the color difference reached the maximum of 4.0 when the concentration of microcapsules was 20.0%, which was significantly higher than that of other core-wall ratios. When the microcapsule concentration was 5.0%, the minimum color difference of the paint film was 1.8. At the 0.67:1 core-wall ratio, the color difference of 20.0% microcapsules reached the maximum of 2.8. The color difference of 0% and 5.0% microcapsules was less than 1.0. At a 0.75:1 core-wall ratio, the color difference of paint films with 0%, 5.0%, and 10.0% microcapsules was less than 1.5, and the fluctuation was relatively small. Except for the paint film without microcapsules, the color difference of paint film with 5.0% microcapsule concentration was the smallest, which reached a minimum of 0.7. In addition, at 20.0% microcapsule concentration, the color difference reached the maximum of 2.6. When the core-wall ratios were 0.67:1 and 0.75:1, the color difference of the paint films with 0–10.0% microcapsules was small and the color was uniform.

The gloss change of paint film can be reflected by irradiating paint film with the same intensity of light from a 60° incident angle. Table 6 reveals the relevance of microcapsule concentration and 60° gloss of paint film. The gloss of the film surface was negatively correlated with the concentration augmentation. As the concentration was augmented from 0 to 20.0%, the gloss lessened from 31.2 G.U. to 1.7 G.U., 1.4 G.U., and 1.8 G.U., respectively. Under the same core-wall ratio, the highest gloss of paint film without microcapsules was 31.2 G.U. After that, with the augmentation of microcapsule concentration, the paint film gloss lessened gradually. Under the condition of a 60° incident angle and the same concentration, the gloss of the 0.75:1 microcapsule was high, slightly better than that of microcapsules with core-wall ratios of 0.42:1 and 0.67:1. Moreover, when the concentration was 5.0% and 10.0% for a 0.75:1 core-wall ratio, the gloss was 11.2 G.U. and 7.8 G.U., respectively. The reason may be that the microcapsules were in the form of powder. When the concentration of microcapsules increased, the particles may have also increased, and the particles would have formed small bumps on the surface of the paint film, which would cause light scattering on the corresponding paint film surface, thus reducing the intrinsic gloss of the paint film [24].

### 3.3. Effect of Microcapsule Concentration on Mechanical Properties of Paint Films

**Figure 4:**
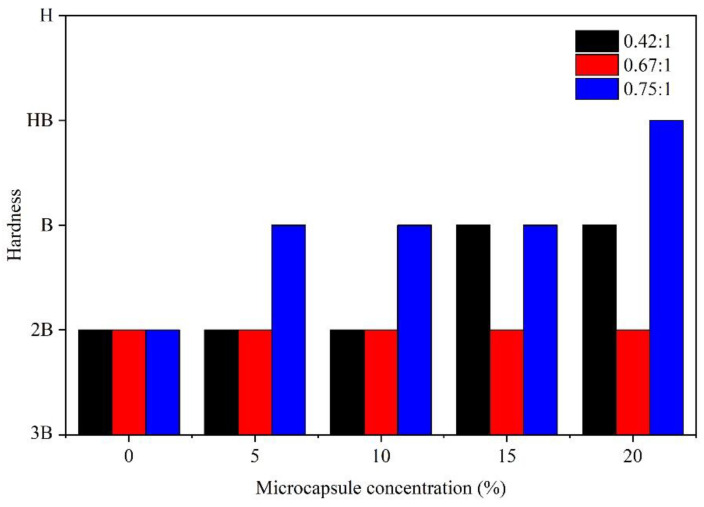
Effect of microcapsule concentration (0.42:1, 0.67:1, and 0.75:1 core-wall ratios) on hardness.

The paint film hardness can reflect the resistance of the paint film to external mechanical action, for instance, collision, as well as scratches, and it is also a significant indicator of the paint film quality. Figure 4 shows the effect of the concentration on paint film hardness. At the 0.42:1 core-wall ratio, as the concentration was augmented from 0% to 15.0%, the hardness was augmented from 2B to B, and the hardness remained at B when the concentration of microcapsules was 20.0%. At the 0.67:1 core-wall ratio, the concentration had no effect on the hardness of the paint films, which were all 2B. At the 0.75:1 core-wall ratio, as the concentration was augmented from 0% to 15.0%, the hardness was augmented from 2B to B. The hardness of the paint film with a 20.0% microcapsule concentration reached the maximum value of HB.

**Figure 5:**
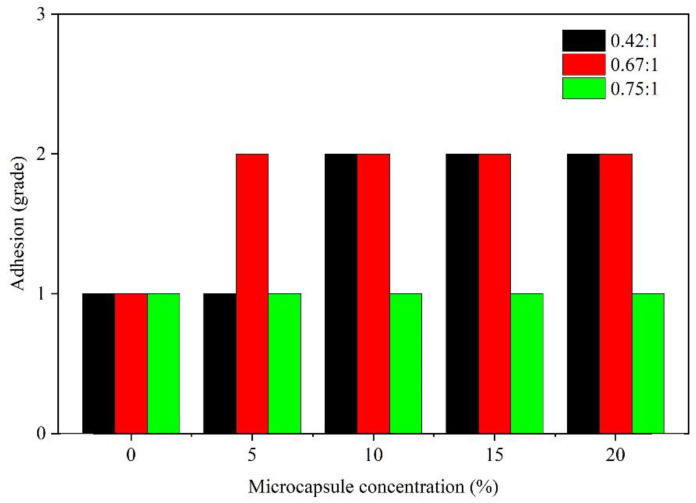
Effect of microcapsule concentration (0.42:1, 0.67:1, and 0.75:1 core-wall ratios) on adhesion.

The adhesion of paint film means the capability of firmly bonding between the paint film and the substrate. The adhesion grade is determined by the damaged area of paint film. The effect of the concentration on the adhesion is indicated in Figure 5. The adhesion of the paint film without microcapsules was good, which was grade 1. At the 0.42:1 core-wall ratio, as the concentration of microcapsules was augmented from 5.0% to 10.0%, the adhesion grade of the paint film lessened from grade 1 to grade 2. At the 0.67:1 core-wall ratio, as the concentration was augmented from 0% to 5.0%, the adhesion grade of the paint film lessened from grade 1 to grade 2. At the 0.75:1 core-wall ratio and under the condition of different microcapsule concentrations, the adhesion grade of the paint film was grade 1, which implies the adhesion was good. The adhesion was not affected by the concentration of microcapsules. This may be because the microcapsules with a 0.75:1 core-wall ratio were more evenly dispersed in the paint film than other core-wall ratios [25].

**Figure 6:**
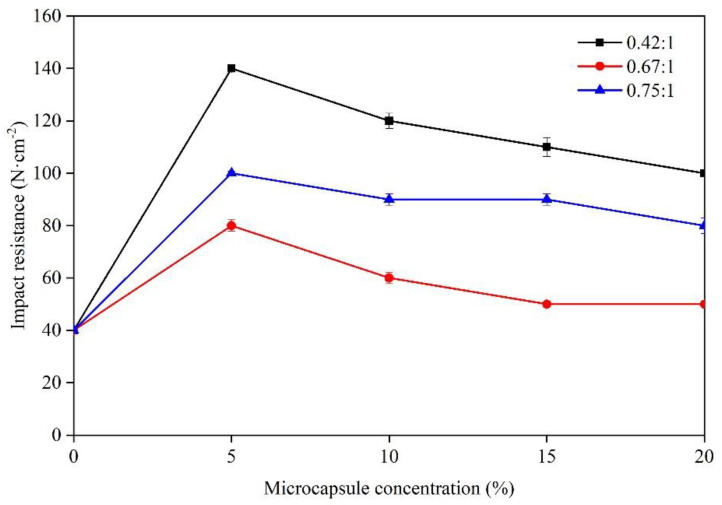
Effect of microcapsule concentration (0.42:1, 0.67:1, and 0.75:1 core-wall ratios) on impact resistance.

Impact resistance means the possibility that the paint film on the substrate may deform under the action of high-speed gravity, but not crack and fall off the substrate. Figure 6 shows that when the concentration of microcapsules (core-wall ratio was 0.42:1, 0.67:1, and 0.75:1) increased from 0% to 5.0%, the impact resistance was good, which was 140 N·cm^−2^, 80 N·cm^−2^, and 100 N·cm^-2^, respectively. After reaching the peak value, the impact resistance gradually lessened with the augmentation of concentration in the range of 5–20.0%. The microcapsule addition could affect the impact resistance of paint film. This may be because the concentration of microcapsules was small, which could enhance the impact resistance [26]. However, with the augmentation of concentration, the paint film surface formed a large agglomeration, leading to the decline of the impact resistance of the paint film. At the 0.42:1 core-wall ratio, the impact resistance was better, compared with the paint film with core-wall ratios of 0.75:1 and 0.67:1 (i.e., 0.42:1 > 0.75:1 > 0.67:1).

### 3.4. Microstructure and Infrared Spectrum Analysis of Waterborne Paint Films with Different Microcapsule Concentrations

**Figure 7:**
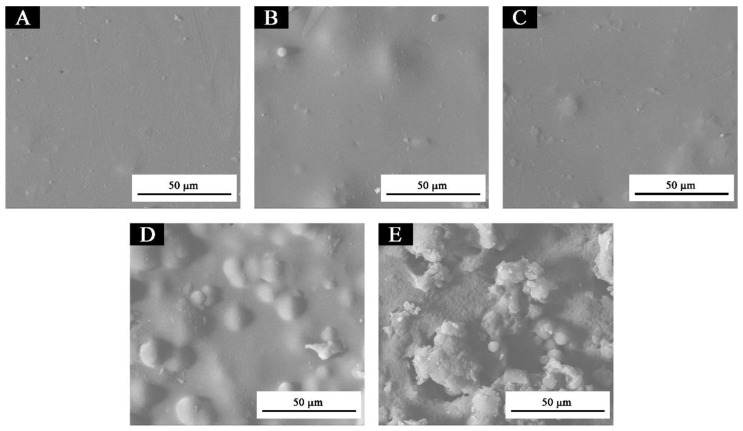
SEM of paint films corresponding to microcapsule (0.75:1 core-wall ratio) concentrations of: (**A**) 0%, (**B**) 5.0%, (**C**) 10.0%, (**D**) 15.0%, (**E**) 20.0%.

**Figure 8:**
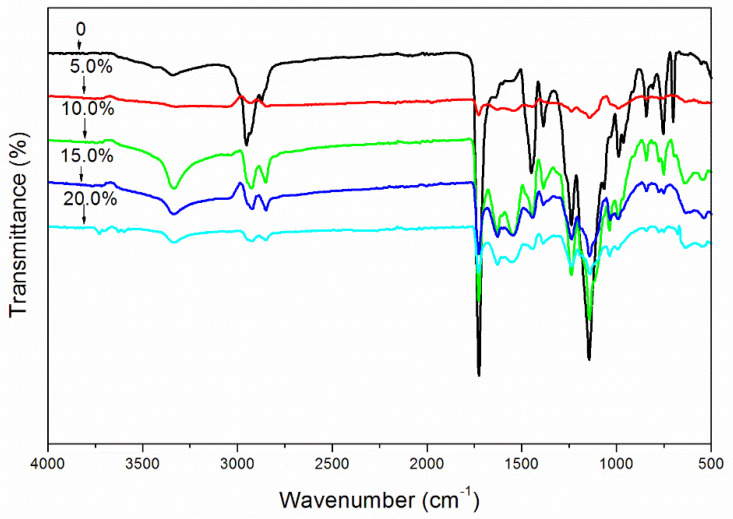
FTIR of paint films corresponding to microcapsule concentration.

Because the microcapsules with a 0.75:1 core-wall ratio had better morphology and structure, smaller color difference, uniform color, and excellent gloss, hardness, and adhesion, the microstructure of the paint film with a 0.75:1 core-wall ratio was analyzed. Figure 7 is the SEM image of the paint film with microcapsules added. The pure paint film (Figure 7A) was smooth and showed almost no particles. The paint film with a 5.0% concentration (Figure 7B) had little small- and medium-sized particle deposition, obvious particles, and no agglomeration. There were some small particles in the paint film (Figure 7C) with a 10.0% concentration, but no agglomeration. When the concentration was augmented to 15.0%, the particles of the waterborne paint film (Figure 7D) increased significantly and agglomerated. The paint film containing 20.0% microcapsules (Figure 7E) had an obvious agglomeration phenomenon. The microstructure of paint films with 5.0% and 10.0% concentrations was better. With the augmentation of the concentration, the number of particles in the paint film also increased, and the particles were more obvious. The increase in microcapsules was not conducive to dispersion, resulting in the agglomeration of microcapsules in the paint film and the weakening of light reflection on the paint film surface, thus reducing the gloss. Figure 8 is the infrared spectrum of paint films corresponding to the addition of different concentrations of microcapsules. The results indicated that the absorption peak of the paint film with 5.0–20.0% microcapsules was consistent with that of the paint film without microcapsules, and no different peaks appear or disappear. Therefore, after the microcapsules were added to the paint film, there was no chemical reaction between the two, only physical binding existed.

### 3.5. Aging and Repair of Waterborne Paint Films

**Figure 9:**
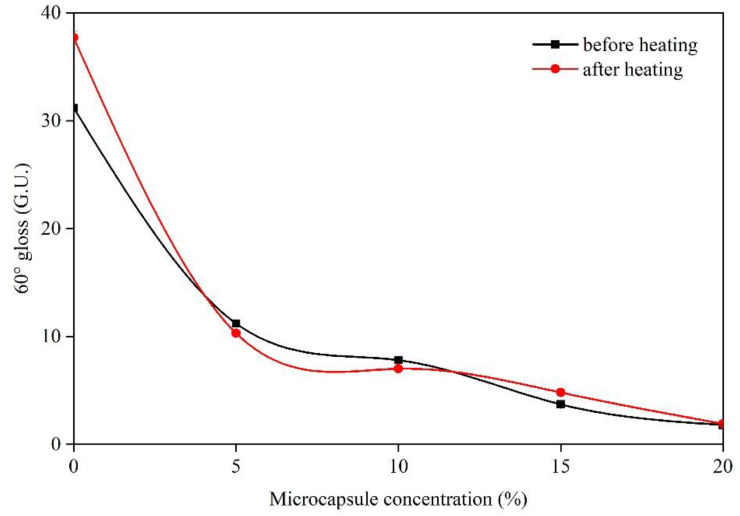
Effect of microcapsule with 0.75:1 core-wall ratio on the paint film gloss.

The self-healing effect of the prepared UF resin-coated shellac resin microcapsules in the aging process of paint films was analyzed by the changes of gloss difference and color difference before and after heating. Table 7 shows the comparison of the gloss difference of paint films before and after heating. Figure 9 shows the influence of microcapsules (core-wall ratio of 0.75:1) on the 60° gloss of paint films before and after heating. The larger the difference of gloss and color difference is, the worse the aging resistance is. According to Table 7 and Figure 9, when the concentration of microcapsules was 5.0%, 10.0%, 15.0%, and 20.0%, the gloss difference before and after heating was small, less than 1.1 G.U. When the concentration of microcapsules was 20.0%, the minimum difference of gloss before and after heating was 0.1 G.U.

Table 8 shows the influence of microcapsules (core-wall ratio was 0.75:1) on the color difference of paint films before and after heating. When the concentration was 10.0% and 15.0%, the color difference before and after heating was 13.3 and 12.8, respectively. When the microcapsule concentration was 10.0%, the values of color difference and gloss changes of the paint film before and after heating were small, and the difference of gloss and the color difference were 0.8 and 13.3, respectively. This might be due to the fact that the microcapsules can repair the microcracks that appeared in the aging process promptly, thus the aging resistance is improved, which implies that the microcapsules with this concentration had the best self-healing effect on the paint film [27].

## 4. Conclusions

UF resin-coated shellac resin microcapsules, with favorable morphology, uniform particle size, and less damage, have been fabricated with a 0.75:1 core-wall ratio. At the 0.42:1 core-wall ratio, when the concentration was 5.0%, the impact resistance was better, which was 140 N·cm^−2^. At the 0.75:1 core-wall ratio, the gloss of paint films with a 5.0% microcapsule concentration was 11.2 G.U. The hardness of paint films with a 20.0% microcapsule concentration reached the maximum value of HB. The adhesion of paint films with microcapsules with a 0.75:1 core-wall ratio was good at different microcapsule concentrations, which were all grade 1. When the concentration of microcapsules was 5.0% and 10.0%, the microstructure of paint films was good. The results revealed that when the core-wall ratio was 0.75:1 and the concentration of microcapsules was 10.0%, the paint film could obtain excellent gloss, hardness, impact resistance, adhesion, and self-healing properties without reducing the original performance of the paint film. At this time, the general properties of the paint film were better, that is, the color difference value was 1.3, the gloss at 60° was 7.8 G.U., adhesion was grade 1, the hardness was B, and the impact resistance was 90 N·cm^−2^. The gloss after the aging process was reduced by 0.8 G.U. and the color difference was 13.3. The results provide the technical reference for the application of self-healing wood paint films.

## Figures and Tables

**Table 1:** The detailed list of each substance dosage for the fabrication of microcapsules.

Sample (#)	Urea (g)	37.0% Formaldehyde Solution (g)	Urea Formaldehyde (UF) Resin (g)	Shellac Resin (g)	Anhydrous Ethanol (g)	Sodium Dodecyl Benzene Sulfonate (SDBS) (g)	Distilled Water (g)	Core-Wall Ratio
1	20.0	27.0	30.0	12.5	62.5	0.975	96.52	0.42:1
2	20.0	27.0	30.0	20.0	100.0	1.560	154.44	0.67:1
3	20.0	27.0	30.0	22.5	112.5	1.760	174.24	0.75:1

**Table 2:** The composition of waterborne topcoat with microcapsules.

Microcapsule Concentration (%)	Microcapsule Weight (g)	Waterborne Topcoat Weight (g)	Self-healing Waterborne Topcoat Weight (g)
0	0	3.00	3.00
5.0	0.15	2.85	3.00
10.0	0.30	2.70	3.00
15.0	0.45	2.55	3.00
25.0	0.60	2.40	3.00

**Table 3:** Effect of microcapsule concentration with 0.42:1 core-wall ratio on the color difference.

Microcapsule Concentration (%)	L_1_	a_1_	b_1_	c_1_	H_1_	L_2_	a_2_	b_2_	c_2_	H_2_	ΔL	Δa	Δb	ΔE
0	71.6 ± 0	13.6 ± 0	36.1 ± 0.1	38.6 ± 0	69.2 ± 0.2	70.4 ± 0.1	14.0 ± 0.1	36.9 ± 0.1	39.5 ± 0.2	69.1 ± 0.2	−1.2 ± 0	0.4 ± 0	0.8 ± 0	1.5 ± 0
5.0	66.7 ± 1.6	14.4 ± 0.4	30.4 ± 0.4	33.7 ± 0.2	64.5 ± 0.2	68.2 ± 1.6	14.8 ± 0.1	31.3 ± 0.2	34.7 ± 0.2	64.6 ± 0.2	1.5 ± 0	0.4 ± 0	0.9 ± 0	1.8 ± 0
10.0	69.8 ± 0.3	13.7 ± 0.1	29.9 ± 0.2	32.9 ± 0.2	65.3 ± 0.2	67.8 ± 0.3	14.6 ± 0.0	29.7 ± 0.7	33.1 ± 0.2	63.8 ± 0.4	−2.0 ± 0	0.9 ± 0	−0.2 ± 0	2.2 ± 0
15.0	63.8 ± 1.2	17.2 ± 0.6	25.1 ± 0.2	30.5 ± 0.3	55.5 ± 1.4	66.1 ± 1.5	17.6 ± 1.5	27.5 ± 0.6	32.7 ± 0.4	57.2 ± 0.6	2.3 ± 0	0.4 ± 0	2.4 ± 0	3.3 ± 0
20.0	71.7 ± 1.2	7.5 ± 0	13.7 ± 0	15.6 ± 0.2	61.3 ± 0	73.8 ± 1.8	9.8 ± 0.2	16.2 ± 0.2	19.0 ± 0.5	58.8 ± 0.4	2.1 ± 0	2.3 ± 0	2.5 ± 0	4.0 ± 0.1

**Table 4:** Effect of microcapsule concentration with 0.67:1 core-wall ratio on the color difference.

Microcapsule Concentration (%)	L_1_	a_1_	b_1_	c_1_	H_1_	L_2_	a_2_	b_2_	c_2_	H_2_	ΔL	Δa	Δb	ΔE
0	74.7 ± 0.2	10.3 ± 0.2	17.2 ± 0.3	20.0 ± 0.4	58.9 ± 0.5	74.7 ± 0.3	10.3 ± 0.2	17.8 ± 0.1	20.0 ± 0.2	58.9 ± 0.7	0 ± 0	0 ± 0	0.6 ± 0	0.6 ± 0
5.0	67.1 ± 0.3	9.8 ± 0.2	21.9 ± 0.2	24.1 ± 0.4	65.7 ± 0.3	67.1 ± 0.2	9.2 ± 0.1	21.5 ± 0.2	24.7 ± 0.3	65.7 ± 0.3	0 ± 0	−0.6 ± 0	−0.4 ± 0	0.7 ± 0
10.0	71.6 ± 0.2	13.6 ± 0.2	36.1 ± 0.2	38.6 ± 0.2	69.2 ± 1.0	70.4 ± 0.4	14.0 ± 0.3	36.9 ± 0.5	39.5 ± 0.5	69.1 ± 1.9	−1.2 ± 0	0.4 ± 0	0.8 ± 0	1.5 ± 0
15.0	77.7 ± 0.2	9.4 ± 0.2	13.4 ± 0.1	16.4 ± 0.5	54.9 ± 0.2	76.4 ± 0.5	10.7 ± 0.2	15.0 ± 0	18.4 ± 0.5	54.5 ± 0.7	−1.3 ± 0	1.3 ± 0	1.6 ± 0	2.4 ± 0
20.0	74.9 ± 0.3	10.6 ± 0.2	18.8 ± 0.3	21.6 ± 0.2	60.5 ± 0.2	77.2 ± 0.2	11.2 ± 0.2	20.3 ± 0.2	23.2 ± 0.2	61.1 ± 0.6	2.3 ± 0	0.6 ± 0	1.5 ± 0	2.8 ± 0

**Table 5:** Effect of microcapsule concentration with 0.75:1 core-wall ratio on the color difference.

Microcapsule Concentration (%)	L_1_	a_1_	b_1_	c_1_	H_1_	L_2_	a_2_	b_2_	c_2_	H_2_	ΔL	Δa	Δb	ΔE
0	68.8 ± 0.2	14.5 ± 0.2	31.0 ± 0.4	34.2 ± 0	64.8 ± 0.5	68.7 ± 0.2	14.2 ± 0.2	31.6 ± 0.2	34.7 ± 0.6	65.8 ± 0.5	−0.1 ± 0	−0.3 ± 0	0.6 ± 0	0.7 ± 0
5.0	66.7 ± 0.4	16.5 ± 0.2	30.1 ± 0.2	34.4 ± 0.3	61.3 ± 0.2	65.5 ± 0.2	16.7 ± 0.4	30.4 ± 0.3	34.7 ± 0.3	61.1 ± 0.3	−1.2 ± 0	0.2 ± 0	0.3 ± 0	1.3 ± 0
10.0	71.6 ± 0.5	13.6 ± 0.2	36.1 ± 0.2	38.6 ± 0.6	69.2 ± 0.6	70.4 ± 0.3	14.0 ± 0	36.9 ± 0.7	39.5 ± 0.2	69.1 ± 0.3	−1.2 ± 0	0.4 ± 0	0.8 ± 0	1.5 ± 0
15.0	70.0 ± 1.7	13.7 ± 0.2	27.9 ± 0.8	31.1 ± 0.7	63.8 ± 0.6	68.5 ± 0.2	14.5 ± 0.2	27.7 ± 0.1	31.3 ± 0.3	62.2 ± 0	−1.5 ± 0	0.8 ± 0	−0.2 ± 0	1.7 ± 0
20.0	74.5 ± 0.3	11.1 ± 0.2	22.5 ± 0	25.1 ± 0.2	63.6 ± 0.4	76.8 ± 0.3	10.9 ± 0.2	23.8 ± 0.2	26.2 ± 0.2	65.3 ± 0.2	2.3 ± 0	−0.2 ± 0	1.3 ± 0	2.6 ± 0

**Table 6:** Effect of microcapsule concentration (0.42:1, 0.67:1, and 0.75:1 core-wall ratios) on 60° gloss.

Microcapsule Concentration (%)	0.42:1	0.67:1	0.75:1
0	31.2 ± 0.2	31.2 ± 0.2	31.2 ± 0.2
5.0	10.5 ± 0.3	9.7 ± 0.2	11.2 ± 0.2
10.0	5.7 ± 0	2.0 ± 0	7.8 ± 0.2
15.0	4.1 ± 0.1	1.9 ± 0	3.7 ± 0.1
20.0	1.7 ± 0	1.4 ± 0	1.8 ± 0

**Table 7:** Effect of microcapsule concentration (0.75:1 core-wall ratio) on 60° gloss.

Microcapsule Concentration (%)	Gloss Before Heating (G.U.)	Gloss after Heating (G.U.)	Difference of Gloss (G.U.)
0	31.2 ± 0.2	37.7 ± 0.6	6.5 ± 0.2
5.0	11.2 ± 0.2	10.3 ± 0.2	0.9 ± 0
10.0	7.8 ± 0.2	7.0 ± 0.1	0.8 ± 0
15.0	3.7 ± 0	4.8 ± 0	1.1 ± 0
20.0	1.8 ± 0	1.9 ± 0	0.1 ± 0

**Table 8:** Effect of microcapsules with 0.75:1 core-wall ratio on the color difference.

Microcapsule Concentration (%)	L_1_	a_1_	b_1_	c_1_	H_1_	L_2_	a_2_	b_2_	c_2_	H_2_	ΔL	Δa	Δb	ΔE
0	62.6 ± 0.2	15.6 ± 0.2	41.8 ± 0.2	44.6 ± 0.5	69.5 ± 0.5	70.4 ± 0.2	14.0 ± 0.2	36.9 ± 0.2	39.5 ± 0.5	69.1 ± 0.5	7.8 ± 0.1	−1.6 ± 0	−4.9 ± 0	9.3 ± 0
5.0	53.1 ± 0	14.0 ± 0.2	32.9 ± 0.2	35.7 ± 0.7	66.9 ± 0.3	68.7 ± 0.2	14.2 ± 0.3	31.6 ± 0.2	34.7 ± 0.2	65.8 ± 0.5	15.6 ± 0.3	0.2 ± 0	−1.3 ± 0	15.7 ± 0.4
10.0	52.6 ± 0.1	13.6 ± 0.2	30.2 ± 0.2	33.2 ± 0.2	65.6 ± 0.5	65.5 ± 0	16.7 ± 0.2	30.4 ± 0	34.7 ± 0.2	61.1 ± 0.2	12.9 ± 0.5	3.1 ± 0	0.2 ± 0	13.3 ± 0.2
15.0	56.0 ± 0.3	12.7 ± 0.2	25.9 ± 0.2	28.8 ± 0.4	63.8 ± 0.6	68.5 ± 0.1	14.5 ± 0.2	27.7 ± 0.2	31.3 ± 0.1	62.2 ± 0.2	12.5 ± 0.2	1.8 ± 0	1.8 ± 0	12.8 ± 0.2
20.0	54.8 ± 0.2	15.8 ± 0.3	22.8 ± 0.2	27.8 ± 0.6	55.2 ± 0	76.8 ± 0.2	10.9 ± 0.2	23.8 ± 0	26.2 ± 0	65.3 ± 0.2	22.0 ± 0.2	−4.9 ± 0.2	1.0 ± 0	22.6 ± 0
